# Mental fatigue does not affect static balance under both single and dual task conditions in young adults

**DOI:** 10.1007/s00221-023-06643-4

**Published:** 2023-05-23

**Authors:** Abubakar Tijjani Salihu, Jibrin Sammani Usman, Keith D. Hill, Maryam Zoghi, Shapour Jaberzadeh

**Affiliations:** 1 Monash Neuromodulation Research Unit, Department of Physiotherapy, School of Primary and Allied Health Care, Faculty of Medicine, Nursing and Health Science, Monash University, Melbourne, Australia; 2 Department of Physiotherapy, Faculty of Allied Health Sciences, Bayero University, Kano, Nigeria; 3 Rehabilitation, Ageing and Independent Living (RAIL) Research Centre, School of Primary and Allied Health Care, Monash University, Frankston, Australia; 4 Discipline of Physiotherapy, Institute of Health and Wellbeing, Federation University Australia, Gippsland, Australia

**Keywords:** Mental fatigue, Static balance, Dual tasking, Young adults

## Abstract

The ability to control balance and prevent falls while carrying out daily life activities may require a predominantly controlled (cognitive) or automatic processing depending on the balance challenge, age, or other factors. Consequently, this process may be affected by mental fatigue which has been shown to impair cognitive abilities. Controlling static balance in young adults is a relatively easy task that may proceed automatically with minimal cognitive input making it insusceptible to mental fatigue. To investigate this hypothesis, static single and dual task (while concurrently counting backward by seven) balance was assessed in 60 young adults (25.2 ± 2.4 years) before and after 45 min of Stroop task (mental fatigue condition) and watching documentary (control), presented in a randomized counterbalanced order on separate days. Moreover, because mental fatigue can occur due to task underload or overload, participants carried out two different Stroop tasks (i.e., all congruent, and mainly incongruent trials) on separate days in the mental fatigue condition. Results of the study revealed a significantly higher feeling of mental fatigue after the mental fatigue conditions compared to control (*p* < 0.001). Similarly, the performance on congruent Stroop trials decreases with time indicating objective mental fatigue (*p* < 0.01). However, there was no difference in balance or concurrent task performance under both single and dual task assessments between the three conditions (*p*  > 0.05) indicating lack of effect of mental fatigue on static balance in this population. Therefore, future studies investigating this phenomenon in occupational or sport settings in similar population should consider using more challenging balance tasks.

## Introduction

The human ability to remain steady while carrying out daily life activities that requires standing, walking, or running depends on a functioning balance control system that constantly keeps the body’s centre of mass within the base of support (Horak 2006). While this system was previously considered to operate automatically under the control of brain stem and spinal cord neuronal circuits (Magnus 1926), further evidence suggests a more complex process that involves higher cortical structures and functions, especially when performing challenging balance tasks or in individuals with underlying balance impairment due to aging or diseases (Horak 2006; Jacobs and Horak 2007; Kerr et al. 1985). For instance, walking down the staircase while carrying a heavy load or standing on a moving bus may require attentional focus to maintain balance and prevent falls even in young adults, while older adults may need to focus attention to maintain their balance under even less challenging circumstances. Therefore, the control of balance is essentially an active process that requires adequate focus of attention under many circumstances (Boisgontier et al. 2013; Takakusaki 2017). The involvement of higher cortical functions including attention and other executive functions in the control of balance implies the susceptibility of this process to mental fatigue, which has been shown to impair various indices of cognitive control (Fletcher and Osler 2021; Holtzer et al. 2010; Van der Linden et al. 2003).

Mental fatigue is a subjective feeling of mental tiredness and inability to sustain attention resulting in performance decrement following prolonged time on cognitive tasks (Chen et al. 2020; Pageaux and Lepers 2018). The neural mechanisms underlining the development of mental fatigue are not completely understood (Van Cutsem et al. 2022). Several theories relating to task overload-induced decrease in motivation (effort-reward imbalance) and neurotoxic waste accumulation or boredom and under arousal resulting from underload have been proposed to explain the negative subjective experience and decreased cognitive performance associated with mental fatigue (Salihu et al. 2022b). From these theories, it can be inferred that both highly demanding (Overload) and monotonous or simple cognitive tasks (Underload) can lead to the development of mental fatigue (Van Cutsem et al. 2022). Regardless of the underlying mechanisms, available evidence indicates a high prevalence of mental fatigue among different occupational and age groups in addition to its pervasiveness among several clinical populations (Fan and Smith 2020; Jang et al. 2021; Ricci et al. 2007; Linnhoff et al. 2019; Lou 2009; Skarpsno et al. 2020). For example, a recent population-based study that included 15,944 vocationally active adults (20–60 years) residing in Nord-Trøndelag County in Norway found that 49% of the sample often experienced mental fatigue, while another 6.5% always feel mentally fatigued after finishing work (Skarpsno et al. 2020). Mental fatigue was also identified to be common among elite athletes in several studies (Russell et al. 2019, 2021, 2022). Similarly, clinical populations such as patients with multiple sclerosis often experiences mental fatigue which is easily exacerbated by cognitive exertion (Linnhoff et al. 2019).

Considering the significant role played by cognition in balance control and the negative effect of mental fatigue on cognitive ability, the high prevalence of mental fatigue in many occupations may put people at risk of injuries related to loss of balance and falls during or after work or sporting events (Lew and Qu 2014). Also, the significant mental fatigue in many clinical populations may affect their ability to control balance potentially leading to fall (Varas-Diaz et al. 2020). In view of this, there is a growing interest in understanding the effect of mental fatigue due to prolonged time performing cognitive tasks on balance in different populations in recent years (Brahms et al. 2022). Several studies investigating the effect of mental fatigue on both static, dynamic, reactive, and proactive balance were carried out in healthy young adults (Deschamps et al. 2013; Fletcher and Osler 2021; Gebel et al. 2022; Hachard et al. 2020; Lew and Qu 2014; Morris and Christie 2020; Qu et al. 2020; Tassignon et al. 2020; Verschueren et al. 2020). However, the findings from these studies are inconsistent with some studies reporting negative effect of mental fatigue on balance (Lew and Qu 2014; Qu et al. 2020) while others found no effect (Fletcher and Osler 2021; Hachard et al. 2020). Since balance performance is task-specific rather than a general ability (Kiss et al. 2018), the lack of effect of mental fatigue on balance in young adults reported in some studies may be related to the type and the degree of complexity of the balance task used.

Indeed, while reactive balance control tasks such as recovery from unexpected perturbations during standing or walking (Lew and Qu 2014; Morris and Christie 2020; Qu et al. 2020) and proactive functional performance balance activities (Tassignon et al. 2020; Verschueren et al. 2020) were consistently impaired by mental fatigue in this population, the same cannot be said for static balance tasks (i.e. where both the base of support and the ground are stationary) which were often unaffected by mental fatigue according to the current available evidence (Deschamps et al. 2013; Fletcher and Osler 2021; Hachard et al. 2020). Obviously, mental fatigue may not have a negative effect on static balance in healthy young adults because they can be able to maintain such types of balance task with minimal cognitive involvement making it insusceptible to cognitive manipulations (Marsh and Geel 2000). However, the current available evidence is inadequate to draw such conclusion for several reasons indicating the need to confirm this in further studies.

Firstly, only three studies investigated the effect of mental fatigue on static balance in healthy young adults and these studies included small number of participants (*n* = 10, *n* = 20, and *n* = 10 respectively) (Deschamps et al. 2013; Fletcher and Osler 2021; Hachard et al. 2020). This suggests the need for further studies with larger sample size adequately powered to detect the true interaction between mental fatigue and static balance in this population. Additionally, often, people carry out static balance tasks concurrently with other cognitive tasks e.g., standing while talking on phone or mentally rehearsing a shopping list. It is therefore ecologically more valid to investigate the effect of mental fatigue on dual task static balance. But only one mental fatigue study in healthy young adults included dual task static balance assessment (Fletcher and Osler 2021). Moreover, while this study included dual task assessment of balance (Fletcher and Osler 2021), it fell short of the essential requirement of reporting on both the balance and cognitive performance during dual tasking (Boisgontier et al. 2013; Salihu et al. 2022a). Therefore, the investigation of the effect of mental fatigue on dual task static balance in this study was incomplete since the changes in the available attentional resources for balance control due to mental fatigue may manifest as a reduction of either the balance performance or the concurrent task performance during dual tasking which was unreported in that study. Finally, since both cognitively demanding and monotonous or simple cognitive tasks can lead to mental fatigue, it is important to investigate the effect of prolonged performance of both types of tasks on static balance. Hence, the aim of this study was to investigate the effect of mental fatigue due to prolonged performance of both cognitive tasks that put high demand on executive control processes and those that are simple and monotonous in nature, on single and dual static balance in healthy young adults. We hypothesized that prolonged performance of both highly demanding and simple monotonous cognitive tasks would result in mental fatigue. But this would not negatively affect static balance tasks performance because the control of these types of balance tasks in young adults can be achieved through a predominantly automatic process. Investigating this hypothesis will highlight the importance of giving due consideration to the controlled/automatic ratio when choosing a balance task for the investigation of the effect of mental fatigue on balance in occupational, clinical or sports settings in a particular population in future studies.

## Methodology

### Participants and sample size calculation

The primary outcome for this study was the participants performance on the modified balance error scoring system (mBESS) assessed using a Food and drugs administration (FDA) approved mobile phone software (Sway medical balance application-SMBA). The total score of the SMBA based mBESS was chosen for an a priori sample size calculation based on previous study by Lee et al. (2017) that indicated the total errors on mBESS were responsive to fatigue in healthy young adults (Lee et al. 2017). Using the data from Amick et al. (2015), the mean baseline total score of the SMBA based mBESS in healthy young adults was 80.0 (SD = 14.37). Assuming an expected intervention (mental fatigue) effect of 3.6 (i.e., 0.25 standard deviation or conventional medium effect size), a sample size of 52 participants (80% power, alpha of 0.05) was required. To have this number of participants complete the study, with an expected dropout rate of 10%, we recruited 60 participants. Accordingly, 60 healthy young adults (49 males, 11 females; mean ± SD; age: 25.2 ± 2.4 years; height: 172.5 ± 7.3 cm; weight: 54.69 ± 9.59 kg) participated in the study. Inclusion criteria were being an undergraduate university student from Bayero University, Kano, Nigeria; and having no known neurological, musculoskeletal, psychiatric, visual or sleep disorder; and not taking any medication that could alter their level of physical or cognitive functions. Written informed consent was obtained from all participants. The study was approved by the local ethics committees of Monash University, Melbourne, Australia (Project ID: 27394) and Bayero University, Kano, Nigeria (NHREC/06/12/19/168).

### Experimental protocol

The study was based on a randomized counterbalanced cross-over design. All experiments took place at the practical demonstration laboratory (Gymnasium), department of physiotherapy, Bayero University, Kano, Nigeria. The participants visited the laboratory four times. The first visit was used to obtain participants’ demographic data (gender, age, height, and weight) and familiarize them with the study procedures. During this visit, the participants received explanation on the study procedures and subjective measurements (mental fatigue visual analogue scale, motivation scale and National Aeronautics and Space Administration (NASA) task load Index) they were expected to fill during their subsequent visits. However, the specific aims and hypotheses of the study were not disclosed to them. Similarly, since they would be required to carry out the Stroop task for induction of mental fatigue, and the balance task during the study, they were also given a practice trial of both tasks (SMBA based mBESS and 120 trials of the Stroop task) during this visit to ensure task familiarization and to mitigate possible learning effects. Subsequently, the order of the experimental and control conditions for each participant was determined at random during this visit. Similarly, the order of the balance assessment i.e., starting with either single or dual task was also randomized with half of the participants starting with single task before dual task and vice-versa.

The remaining three visits were allocated to the experimental (induction of mental fatigue, under two conditions) and one control (watching a documentary) condition. Adequate wash-out period (at least 7 days) was allowed between the three sessions to avoid carry-over effects. Participants attended the laboratory at the same time of the day for all conditions to control for circadian effects. They were also instructed to ensure adequate sleep (at least 7 h) the night before; refrain from consumption of caffeine or alcohol 12 h before; and not to engage in rigorous physical exercise 24 h before each of the testing sessions. Participants were interviewed with a checklist of these instructions before each condition to ensure strict compliance. Each condition involved recording the participants single and dual task balance performance using the SMBA based mBESS pre-intervention. During the dual task assessment, the participants completed the balance task while concurrently counting backward by seven from a random number selected by the assessor between 200 and 300. The participants also completed the cognitive task of counting backward by seven for the same duration as the balance task in a seated position as a single task. This and the dual task balance assessment were video recorded, and the total numbers counted backward (both correct and incorrect) were analysed offline.

**Fig. 1:**
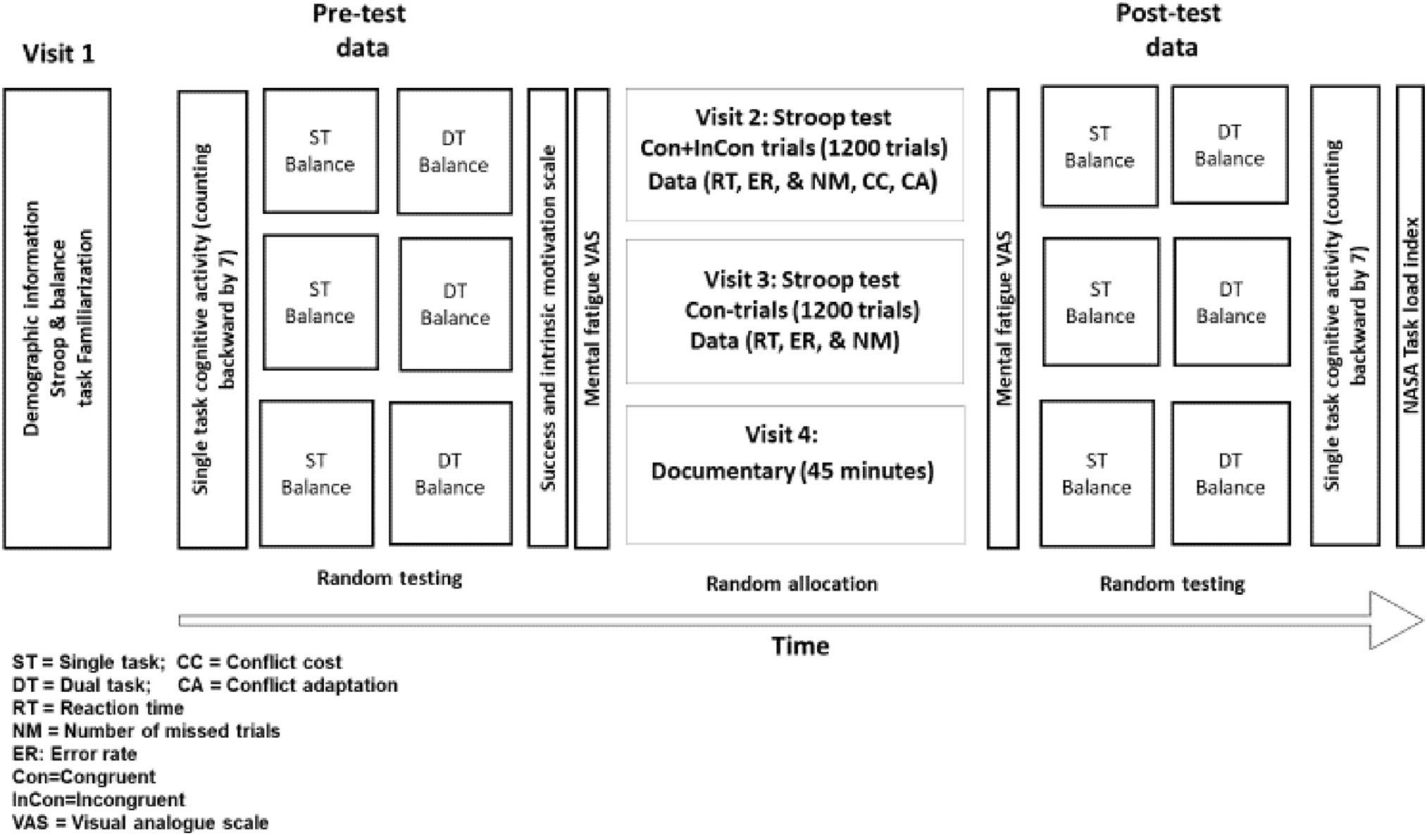
Experimental protocol

The balance assessment tool and procedure were explained in detail in the outcome measures section below. After the balance assessment, the participants were seated comfortably and asked to fill the questionnaire on motivation and mental fatigue visual analogue scale before performing the Stroop task or watching the documentary. Immediately after completion of the task (Stroop task or watching documentary), they were asked to fill the mental fatigue visual analogue scale again before repeating the balance assessment as done in the pre-test condition. They were then asked to complete the National Aeronautics and Space Administration Task Load Index (NASA) task load index that asked about the perceived workload of the completed experimental or control conditions (Fig. 1).

### Mental fatigue and control tasks

We used two different Stroop tasks in the two experimental conditions to test our hypothesis of mental fatigue caused by prolonged performance of cognitively demanding or simple monotonous tasks. The first Stroop task (Stroop task_Overload_) contains a majority of incongruent trials (75%). Performance of incongruent Stroop trials requires selective attention and response inhibition thereby putting considerable demand on the executive control process (Mansouri et al. 2009; Van Cutsem et al. 2022). Accordingly, this Stroop task may fit into the overload hypothesis of mental fatigue mechanism. On the other hand, our second Stroop task (Stroop task_Underload_) contain 100% congruent trials. Responding to congruent colour-word stimuli does not require response inhibition and selective attention like the incongruent ones. Rather, it only involves simple detection and discrimination of stimuli and may thus cause mental fatigue based on the underload hypothesis (Langner and Eickhoff 2013; Pattyn et al. 2008). Both Stroop tasks contained 1200 trials presented continuously without break. In each trial, one of four coloured words (“red”, “yellow”, “green”, or “blue”) was presented on a computer screen for 2000 ms and if the participant was unable to respond within this time frame, the trial was automatically recorded as a missed trial. Otherwise, the time between the presentation of the stimulus and participant’s response was automatically recorded as the reaction time for that trial. For incongruent trials, the meaning of the word and the colour of the ink it was printed were different (e.g., the word “green” printed in “red” ink) and participants were asked to ignore the meaning of the word and respond based on the colour of the ink by pressing the letter that correspond to the first letter of that colour on the computer keyboard (e.g., “r” for red or “g” for green). In contrast, the meaning of the word and the colour of the ink it was printed in were the same in the congruent trials and participants responded by pressing the letter that correspond to the presented coloured word on the computer keyboard. In each case, incorrect responses were recorded as errors, and feedback indicating correct or incorrect response appeared on the computer screen after each trial before the next trial was presented. Participants were instructed to respond as quickly and accurately as possible. Both Stroop tasks were conducted using the Psytoolkit platform. This is a free-to-use toolkit for demonstrating, programming, and running cognitive experiments (Stoet 2010, 2017).

During the control condition, the participants watched a documentary (“When we left earth-the NASA missions” by Discovery entertainments, 2008) for 45 min. The contents of this documentary are both engaging and emotionally neutral justifying its choice in the current and several previous mental fatigue studies (Van Cutsem et al. 2019, 2022; Verschueren et al. 2020). The duration of the documentary was based on our pilot study (data not presented) that shows that the participants were able to complete the 1200 trials of the Stroop task in around 45 min.

### Outcome measures

#### Balance performance

Balance was assessed using the modified balance error scoring system (mBESS) test using the sway medical balance application (SMBA) (Sway Medical, Tulsa, OK, USA). SMBA is an FDA approved mobile device software application which, when installed on a mobile consumer electronic device, accesses the micro electromechanical system (MEMS) tri-axial accelerometer output to assess balance by calculating postural sway (Dabbs et al. 2017). The SMBA based mBESS protocol consists of five stances including bipedal (feet together), tandem stance (left foot forward), tandem stance (right foot forward), single leg stance (right), and single leg stance (left). For the current study, the SMBA was used on a Tecno camon 12 mobile phone (Tecno mobile, Shenzhen, China) and participants performed each stance twice on a firm surface with eyes closed for a period of 10 s and the average score of the two tests was recorded. For the duration of each stance, the subject held the measuring device upright against the mid-point of their sternum with both hands. The SMBA software calculated a score for each stance as well as a total score on a scale from 0 to 100 with higher scores indicating better postural stability. The SMBA based mBESS scores has moderate to excellent test–retest reliability (Amick et al. 2015; Mummareddy et al. 2020).

### Concurrent task (counting backward by seven) performance

Accuracy ratio (number of correct responses/number of total responses) was calculated and then multiplied by 100 to get the percentage of correct responses in the counting backward task.

### Subjective mental fatigue

To measure subjective feeling of mental fatigue, the mental fatigue visual analogue scale (M-VAS) was used. This is a horizontal rating scale with a fixed length (100 mm) and two anchors that range from 0 (not at all mentally fatigued) to 100 (extremely mentally fatigued) (Chen et al. 2020; Herlambang et al. 2019). This scale has good test–retest reliability and validity to measure subjective fatigue (Herlambang et al. 2019).

### Objective measurement of mental fatigue

To objectively assess mental fatigue in the participants, we examined the time-on-task effect on the Stroop tasks by dividing the 1200 trials into four blocks of 300 trials each during the analysis. Mean reaction time, error rates and number of missed trials were calculated and compared across the four blocks to obtain an objective index of mental fatigue in the two Stroop tasks. For the Stroop task_Overload_, in addition to the absolute reaction time and number of errors or missed trials in each block, we also calculated the conflict cost and conflict adaptation. Conflict cost is the difference in reaction time between the incongruent (high conflict) and the congruent (low conflict) Stroop trials (Mansouri et al. 2009). On the other hand, conflict adaptation is the facilitative effect of previously experienced conflict whereby the reaction time (RT) or error rate in high-conflict trials that are immediately preceded by another high-conflict trial (HH condition) are lower than those in high-conflict trials that are immediately preceded by a low-conflict trial (LH condition) (Mansouri et al. 2009). The conflict adaptation in RT was calculated by subtracting the HH RT from the LH RT. Conversely, the conflict adaptation in percentage of correct responses (% correct) was calculated by subtracting the % correct in LH from HH.

### Motivation

Participants’ motivation towards the upcoming tasks (Stroop tasks or documentary) was assessed using the success and intrinsic motivation scale developed and validated by Matthews et al. (2001), Verschueren et al. (2020). This scale involves a response to questions related to success and intrinsic motivations towards the upcoming task with a range of possible scores between 0 and 32 and 0and 28 for the success and intrinsic motivation, respectively. Participants completed this scale before the start of each condition.

### Perceived workload

The National Aeronautics and Space Administration Task Load Index (NASA-TLX) was completed by the participants at the end of each condition to assess the level of perceived workload associated with that condition. The NASA-TLX assesses six aspects of perceived workload about the experimental or control intervention, namely: “mental demand, physical demand, temporal demand, performance, effort and frustration” (Hart and Staveland 1988). Each of the six subscales of the NASA-TLX is scored on a scale between 0 and 100.

### Statistical analysis

Statistical analyses were conducted using the Statistical Package for the Social Sciences (SPSS) version 28 (IBM Corp., Armonk, NY, USA). Significance was set at 0.05 and the descriptive data were presented as mean and standard deviation (SD) except where stated otherwise. The data were first screened for normal distribution using the Shapiro–Wilk test and visual inspection of histograms.

The participants motivation towards the upcoming task (Stroop task_Overload_, Stroop task_Underload_ and the control task) were compared using one-way repeated measure analysis of variance (RM-ANOVA). Similar one-way RM-ANOVA was used to compare the participant’s perceived workload on each of the NASA-TLX subscales after the three conditions. The change in conflict cost and conflict adaptation with time during the Stroop task_Overload_ was also analysed using one-way RM-ANOVA. The data on the reaction time, error rate and the number of missed trials during the Stroop task_Underload_ was not normally distributed even after square root transformation. Therefore, to test the effect of time (blocks) on this data, nonparametric Freidman test was used. If significant main effect of block was found, Wilcoxon signed rank tests with Bonferroni correction were conducted to compare the different blocks.

Two-way (2 × 4) RM-ANOVA was conducted to test the effect of congruency (congruent and incongruent trials) and time (block 1 to block 4) on the reaction time, number of errors and number of missed trials during the Stroop task_Overload_. Likewise, to test the effect of time (pre and post) and condition (Stroop task_Overload_, Stroop task_Underload_ and the control task) on subjective feeling of mental fatigue, a two-way (2 × 3) RM-ANOVA was applied to the M-VAS scores of the participants. Finally, three-way (2 × 2 × 3) RM-ANOVA was conducted to test the effect of time (pre and post), type of balance task (single or dual task) and experimental condition (Stroop task_Overload_, Stroop task_Underload_ and the control task) on each of the balance outcomes. Similar three-way (2 × 2 × 3) RM-ANOVA was applied to the concurrent task (counting backward by seven) data to test the effect of time (pre and post), type of task (single or dual task) and experimental condition (Stroop task_Overload_, Stroop task_Underload_ and the control task).

Before interpreting RM ANOVA statistical outcomes, sphericity was verified by Mauchly’s test. When the assumption of sphericity was violated, Greenhouse-Geiser corrected significance values were used. If RM ANOVA showed significant main or interaction effects, the main and simple main effects were further investigated using post-hoc pairwise comparisons with Bonferroni correction.

## Result

We encountered some missing data in a small number of participants for some outcome measures (Subjective mental fatigue: 3 participants; Stroop task_Underload_: 2 participants; NASA TLX Index: 1 participant; Intrinsic motivation: 4 participants) due to computer technical problems (Stroop task result transmission) and lost data records (other outcome measures). Therefore, the results in such cases were based on the remaining participants where we have the complete data.

### Task motivation and perceived workload

Participants did not show significant difference in intrinsic motivation (*F*(2,118) = 2.737, *p* = 0.069, $\eta_{{\text{p}}}^{2}$ = 0.044) and motivation for task success (*F*(2,118) = 0.302, *p* = 0.740, $\eta_{{\text{p}}}^{2}$ = 0.005) between the experimental and control conditions. On the other hand, NASA-TLX data showed that the participants perceived the performance of both Stroop tasks as more physically (*F*(2,116) = 5.999, *p* = 0.003, $\eta_{{\text{p}}}^{2}$ = 0.094) and temporally demanding (*F*(1.667,96.672) = 11.029, *p* < 0.001, $\eta_{{\text{p}}}^{2}$ = 0.160), as well as more frustrating than the control task (*F*(2,116) = 9.170, *p* < 0.001, $\eta_{{\text{p}}}^{2}$ = 0.137) (Table 1). However, only the performance of Stroop task_Overload_ was perceived as more mentally demanding (*F*(2,114) = 5.087, *p* = 0.008, $\eta_{{\text{p}}}^{2}$ = 0.082; post hoc comparison: Stroop task_Overload_ versus control, *p* = 0.012; other pairs, *p* ˃ 0.05) and effortful than the control task (*F*(1.709,99.101) = 4.271, *p* = 0.022, $\eta_{{\text{p}}}^{2}$ = 0.069; post hoc comparison: Stroop task_Overload_ versus control, *p* = 0.039; other pairs, *p* >  0.05) (Table 1). There was no significant difference in the performance subscale of the NASA-TLX between the three conditions (*F*(2,116) = 1.769, *p* = 0.175, $\eta_{{\text{p}}}^{2}$ = 0.030). Similarly, the two Stroop tasks did not differ significantly in any of the NASA-TLX subscales (Table 1).

**Table 1:** Questionnaires outcome

Questionnaire	Stroop task_Overload_	Stroop task_Underload_	Documentary
Intrinsic motivation	11.850 (3.246)	12.483 (2.999)	12.700 (3.386)
Task success motivation	19.067 (4.697)	19.300 (5.324)	19.483 (5.376)
NASA-TLX			
Mental demand	64.74 (23.776)*	60.345 (21.396)	53.966 (24.723)
Physical demand	56.356 (23.759)*	57.288 (23.985)*	46.441 (27.277)
Temporal demand	60.169 (22.399)*	57.712 (19.725)*	45.509 (24.489)
Performance	40.085 (25.146)	40.339 (25.979)	33.898 (23.998)
Effort	62.797 (22.424)*	61.186 (20.623)	53.729 (24.941)
Frustration	41.017 (26.809)*	38.559 (23.101)*	25.763 (21.830)

Note: Data are presented as mean ± SD

*Significantly different from the control condition (Watching documentary)

### Indicators of mental fatigue

**Fig. 2:**
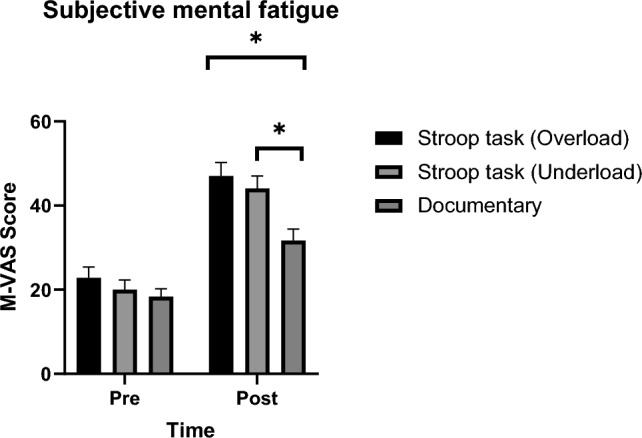
Subjective feeling of mental fatigue. *Indicate a significant difference between the experimental and control condition. Data is presented as mean and SE

A significant interaction between experimental condition and time was found in the participant’s subjective feeling of mental fatigue (*F*(2,112) = 6.175, *p* = 0.003, $\eta_{{\text{p}}}^{2}$ = 0.099). Post hoc pairwise comparison revealed that the subjective feeling of mental fatigue increases from pre to post in all the three conditions. However, participants feel more mentally fatigued after completing both Stroop tasks compared to the control condition (*p* < 0.001) (Fig. 2). There was no significant difference in subjective feelings of mental fatigue between the two Stroop tasks post intervention.

**Fig. 3:**
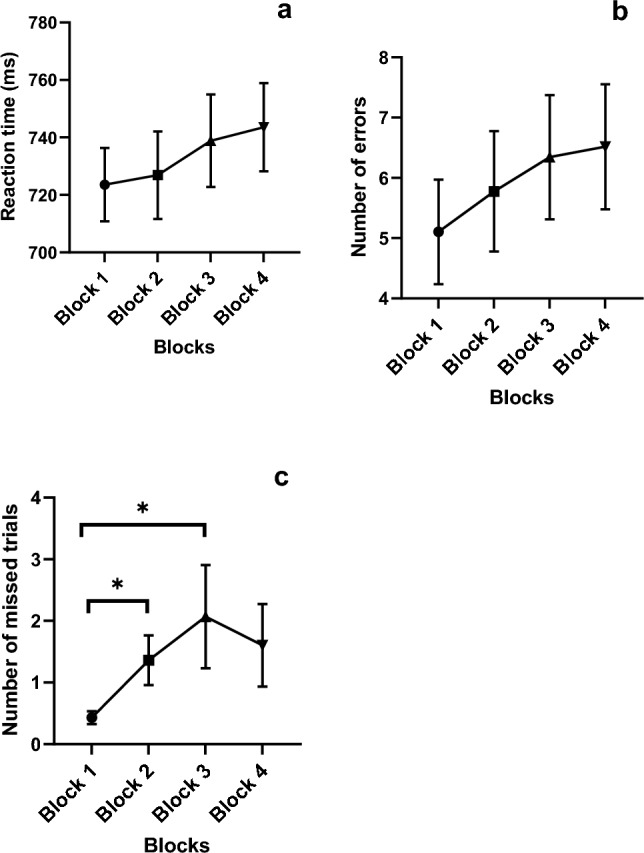
**a**–**c** Objective assessment of mental fatigue during the Stroop task_Underload_. *Indicates a significant difference between blocks. Data is presented as mean and SE

**Fig. 4:**
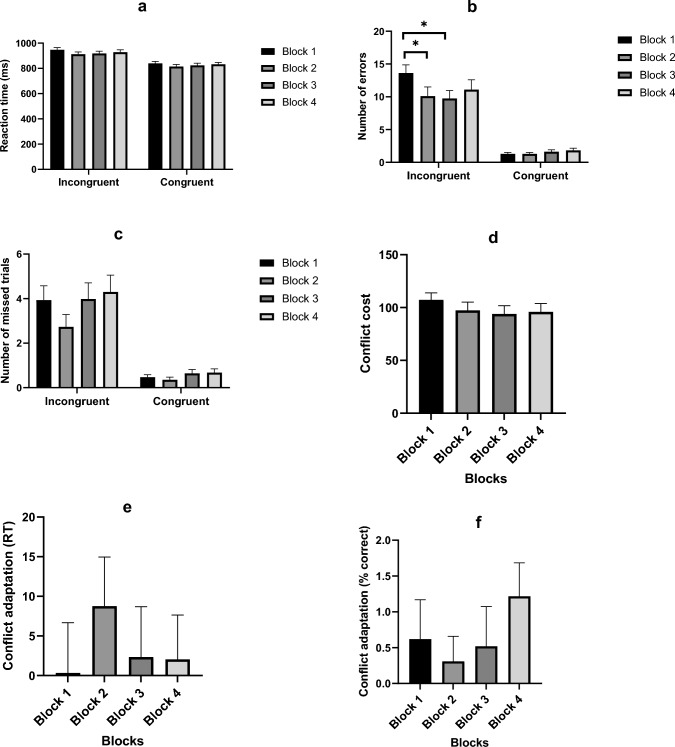
**a**–**f** Objective assessment of mental fatigue during the Stroop task_Overload_. *Indicates a significant difference between blocks. *RT* reaction time. Data are presented as mean and SE

Regarding the objective markers of mental fatigue, there was no significant main effect of block on the reaction time and error rate during the Stroop task_Underload_ (*p* ˃ 0.05). For the number of missed trials during this task, there was a significant main effect of blocks (*χ*^2^(3) = 11.58, *p* = 0.009). Post hoc comparison revealed that this was due to a significantly higher number of missed trials in blocks 2 (*p* = 0.003) and 3 (*p* = 0.007) compared to block 1 (Fig. 3c). There was no effect of blocks on both the conflict cost (*F*(3,177) = 1.394, *p* = 0.246, $\eta_{{\text{p}}}^{2}$ = 0.023) and conflict adaptation (RT: *F*(3,177) = 0.365, *p* = 0.778, $\eta_{{\text{p}}}^{2}$ = 0.006; % correct: *F*(3,177) = 0.624, *p* = 0.600, $\eta_{{\text{p}}}^{2}$ = 0.010) during performance of the Stroop task_Overload_. However, the two-way repeated measure ANOVA revealed a significant interaction between congruency and blocks in the number of errors during the performance of this task (*F*(2.643,155.929) = 7.569, *p* < 0.001, $\eta_{{\text{p}}}^{2}$ = 0.114). Post-hoc pairwise comparison showed that the number of errors significantly decreased in blocks 2 and 3 compared to block 1 during the incongruent trials (*p* < 0.05) (Fig. 4b). The number of errors were also significantly higher during the incongruent compared to congruent trials in all the blocks (*p* < 0.001). Analysis of both the reaction time and the number of missed trials during performance of the Stroop task_Overload_ did not show significant interaction between blocks and congruency (Reaction time: *F*(3,177) = 1.393, *p* = 0.246, $\eta_{{\text{p}}}^{2}$ = 0.023; number of missed trials: *F*(2.169,125.830) = 1.615, *p* = 0.201, $\eta_{{\text{p}}}^{2}$ = 0.027). But there were significant main effects of both blocks (Reaction time: *F*(2.232,131.662) = 3.85, *p* = 0.02, $\eta_{{\text{p}}}^{2}$ = 0.061; number of missed trials: *F*(2.551,147.98) = 3.07, *p* = 0.037, $\eta_{{\text{p}}}^{2}$ = 0.050 and congruency (Reaction time: *F*(1,59) = 250.2, *p* < 0.001, $\eta_{{\text{p}}}^{2}$ = 0.81; number of missed trials: *F*(1,58 = 30.74, *p* = *p* < 0.001, $\eta_{{\text{p}}}^{2}$ = 0.346) on both outcomes. Post hoc comparison revealed that the main effect of blocks on the reaction time was because averaged across both congruent and incongruent trials, the reaction time was lower in block 2 compared to block 1 (*p* = 0.004). For the main effect of blocks on the number of missed trials, post hoc comparison showed that this was because averaged across both congruent and incongruent trials, the number of missed trials was higher in block 4 compared to block 2 (0.040). Finally, the main effect of congruency was basically because averaged across all blocks, both the reaction time and number of missed trials were higher in the incongruent than congruent Stroop trials (*p* < 0.05).

### Balance performance

The result of the statistical analysis of the balance outcome measures has been summarized in Tables 2, 3. There was no significant three-way interaction between experimental conditions (Stroop task_Overload_, Stroop task_Underload_ and control), time (pre and post) and task (single and dual) for any of the balance outcomes. Similarly, there was no significant two-way interaction between experimental condition and time for any of the balance outcomes. There was, however, a significant two-way interaction between experimental conditions and task during single leg stance on the right lower limb (*F*(2,118) = 5.59, *p* = 0.005, $\eta_{{\text{p}}}^{2}$ = 0.086) and in total balance score (*F*(2,118) = 6.40, *p* = 0.002, $\eta_{{\text{p}}}^{2}$ = 0.098). Post hoc comparison showed that this interaction was because averaged across time (pre and post), dual task performance for these two balance outcomes was significantly lower than single task but only in the two mental fatigue conditions (*p* < 0.05). Similarly, there was a significant main effect of experimental condition during performance of balance task with feet together (*F*(2,118) = 3.521, *p* = 0.03, $\eta_{{\text{p}}}^{2}$ = 0.056) and in total balance score (*F*(1.78,105.08) = 3.95, *p* 0.026, $\eta_{{\text{p}}}^{2}$ = 0.063). Post hoc pairwise comparison revealed that this stemmed from the lower balance score averaged across both time (pre and post) and type of task (single and dual) in the Stroop task_Overload_ condition compared to the control condition while standing with feet together (*p* = 0.015). None of the pairs of comparison in the total balance score reach statistical significance (*p* > 0.05). Lastly, there was a significant main effect of task (single or dual) for all the balance tests. This is because averaging across both experimental conditions and time, the participants’ balance performance was better under single compared to the dual task condition (*p* < 0.05).

**Table 2:** Descriptive data-balance outcome

Balance tests	Experimental conditions											
	STROOP TASK_Overload_				STROOP TASK_Underload_				DOCUMENTARY			
	PRE		POST		PRE		POST		PRE		POST	
	Single task Mean (SD)	Dual task Mean (SD)	Single task Mean (SD)	Dual task Mean (SD)	Single task Mean (SD)	Dual task Mean (SD)	Single task Mean (SD)	Dual task Mean (SD)	Single task Mean (SD)	Dual task Mean (SD)	Single task Mean (SD)	Dual task Mean (SD)
FT (EC)	98.418 (2.024)	95.761 (4.195)	98.074 (1.887)	95.496 (4.332)	98.601 (1.624)	96.269 (3.890)	98.457 (2.310)	95.226 (6.347)	98.669 (1.766)	98.284 (3.585)	98.901 (1.589)	96.799 (3.598)
TR (EC)	95.788 (4.204)	92.572 (6.721)	95.126 (4.513)	92.213 (8.598)	95.957 (3.889)	92.153 (9.440)	96.292 (3.442)	91.781 (8.737)	95.119 (7.661)	92.275 (10.249)	95.212 (5.165)	94.052 (7.115)
TL (EC)	95.312 (7.341)	90.809 (10.137)	94.879 (6.553)	90.606 (10.394)	94.619 (6.933)	92.065 (8.113)	95.205 (6.295)	91.324 (9.557)	96.133 (6.718)	92.719 (7.664)	95.751 (7.002)	91.698 (9.654)
SL-R EC)	80.079 (20.679)	76.541 (23.802)	82.421 (23.751)	75.261 (26.892)	78.828 (21.961)	75.173 (23.143)	81.368 (20.436)	75.733 (27.580)	77.594 (21.873)	78.117 (21.666)	79.058 (22.048)	81.138 (21.126)
SL-L (EC)	76.691 (24.207)	74.256 (25.553)	76.711 (24.981)	71.740 (28.281)	78.922 (21.489)	77.004 (23.148)	78.773 (23.636)	70.537 (29.165)	78.615 (23.592)	76.638 (22.601)	80.820 (20.992)	78.321 (24.540)
Total	89.554 (8.910)	84.742 (13.527)	89.300 (9.816)	84.992 (13.068)	89.342 (8.720)	86.138 (11.632)	90.208 (8.467)	84.833 (13.771)	89.567 (8.604)	87.768 (9.542)	89.900 (10.054)	88.783 (10.791)

**Table 3:** Statistics-balance outcome

Balance tests	Main and interaction effects						
	Three-way interaction	Two-way interactions			Main effects		
	Exp-cond × time × task	Exp-cond × time	Exp-cond × task	Time × task	Exp-cond	Time	Task
FT (EC)	(*F*(1.79,105.35) = 1.36, *p* = 0.261, $\eta_{{\text{p}}}^{2}$ = 0.023	(*F*(1.83,108.3) = 2.98, *p* = 0.059, $\eta_{{\text{p}}}^{2}$ = 0.048	(*F*(1.7,97.9) = 0.65, *p* = 0.49, $\eta_{{\text{p}}}^{2}$ = 0.011	(*F*(1,59) = 0.23, *p* = 0.64, $\eta_{{\text{p}}}^{2}$ = 0.004	(*F*(2,118) = 3.521, *p* = 0.03*, $\eta_{{\text{p}}}^{2}$ = 0056	(*F*(1,59) = 0.735, *p* = 0.39, $\eta_{{\text{p}}}^{2}$ = 0.012	(*F*(1,59) = 48.95, *p* = 0.000*, $\eta_{{\text{p}}}^{2}$ = 0.453
TR (EC)	(*F*(2,118) = 1.07, *p* = 0.35, $\eta_{{\text{p}}}^{2}$ = 0.018	(*F*(2,118) = 1.29, *p* = 0.28, $\eta_{{\text{p}}}^{2}$ = 0.021	(*F*(1.84,108.29) = 2.05, *p* = 0.14, $\eta_{{\text{p}}}^{2}$ = 0.034	(*F*(1,59) = 0.37, *p* = 0.54, $\eta_{{\text{p}}}^{2}$ = 0.006	(*F*(2,118) = 0.081, *p* = 0.923, $\eta_{{\text{p}}}^{2}$ = 0.001	(*F*(1,59) = 0.113, *p* = 0.74, $\eta_{{\text{p}}}^{2}$ = 0.002	(*F*(1,59) = 25.38, *p* = 0.000*, $\eta_{{\text{p}}}^{2}$ = 0.301
TL (EC)	(*F*(2,118) = 0.30, *p* = 0.74, $\eta_{{\text{p}}}^{2}$ = 0.005	(*F*(2,118) = 0.21, *p* = 0.82, $\eta_{{\text{p}}}^{2}$ = 0.003	(*F*(1.85,109.38) = 0.57, *p* = 0.56, $\eta_{{\text{p}}}^{2}$ = 0.010	(*F*(1,59) = 0.43, *p* = 0.52, $\eta_{{\text{p}}}^{2}$ = 0.007	(*F*(1.81,106.79) = 1.58, *p* = 0.21, $\eta_{{\text{p}}}^{2}$ = 0.026	(*F*(1,59) = 0.71, *p* = 0.40, $\eta_{{\text{p}}}^{2}$ = 0.012	(*F*(1,59) = 26.53, *p* = 0.000*, $\eta_{{\text{p}}}^{2}$ = 0.310
SL-R (EC)	(*F*(2,118) = 0.45, *p* = 0.64, $\eta_{{\text{p}}}^{2}$ = 0.007	(*F*(2,118) = 0.20, *p* = 0.82, $\eta_{{\text{p}}}^{2}$ = 0.003	(*F*(2,118) = 5.59, *p* = 0.005*, $\eta_{{\text{p}}}^{2}$ = 0.086	(*F*(1,59) = 0.49, *p* = 0.48, $\eta_{{\text{p}}}^{2}$ = 0.008	(*F*(2,118) = 0.29, *p* = 0.75, $\eta_{{\text{p}}}^{2}$ = 0.005	(*F*(1,59) = 1.21, *p* = 0.28, $\eta_{{\text{p}}}^{2}$ = 0.020	(*F*(1,59) = 4.91, *p* = 031*, $\eta_{{\text{p}}}^{2}$ = 0.077
SL-L (EC)	(*F*(2,118) = 0.69, *p* = 0.50, $\eta_{{\text{p}}}^{2}$ = 0.012	(*F*(2,118) = 1.78, *p* = 0.17, $\eta_{{\text{p}}}^{2}$ = 0.029	(*F*(2,118) = 0.72, *p* = 0.49, $\eta_{{\text{p}}}^{2}$ = 0.012	(*F*(1,59) = 2.69, *p* = 0.11, $\eta_{{\text{p}}}^{2}$ = 0.044	(*F*(2,118) = 2.74, *p* = 0.069, $\eta_{{\text{p}}}^{2}$ = 0.044	(*F*(1,59) = 0.47, *p* = 0.49, $\eta_{{\text{p}}}^{2}$ = 0.008	(*F*(1,59) = 7.87, *p* = 0.007*, $\eta_{{\text{p}}}^{2}$ = 0.118
Total	(*F*(2,118) = 1.38, *p* = 0.26, $\eta_{{\text{p}}}^{2}$ = 0.023	(*F*(2,118) = 0.39, *p* = 0.68, $\eta_{{\text{p}}}^{2}$ = 0.007	(*F*(2,118) = 6.40, *p* = 0.002*, $\eta_{{\text{p}}}^{2}$ = 0.098	(*F*(1,59) = 0.23, *p* = 0.64, $\eta_{{\text{p}}}^{2}$ = 0.004	(*F*(1.78,105.08) = 3.95, *p* 0.026*, $\eta_{{\text{p}}}^{2}$ = 0.063	(*F*(1,59) = 0.09, *p* = 0.76, $\eta_{{\text{p}}}^{2}$ = 0.002	(*F*(1,59) = 29.91, *p* = 0.000*, $\eta_{{\text{p}}}^{2}$ = 0.305

*EC* eyes closed, *FT* standing with feet together, *TR* tandem stance with right foot forward, *TL* tandem stance with left foot forward, *SL-R* single leg stance on the right foot, *SL-L* single leg stance on the left foot

Regarding the effect on the performance of the counting backward task, there was no significant three-way interaction between experimental conditions (Stroop task_Overload_, Stroop task_Underload_ and control), time (pre and post) and task (single and dual) for any of the balance outcomes (Tables 4, 5). Similarly, none of the two-way interactions reached statistical significance. There was, however, a significant main effect of task while counting backwards by seven in a single leg stance on the right lower limb (*F*(1,59) = 7.79, *p* = 0.007, $\eta_{{\text{p}}}^{2}$ = 0.117). Post hoc pairwise comparison revealed that this was because, averaged across both time (pre and post) and experimental conditions, the performance of the counting backward task was lower during dual tasking in this stance compared to single task.

**Table 4:** Descriptive data-counting backward task (data is presented as percentage of correct responses i.e., accuracy ratio × 100)

Balance tests	Experimental conditions											
	Experimental Stroop				Control Stroop				Control documentary			
	PRE		POST		PRE		POST		PRE		POST	
	Single task Mean (SD)	Dual task Mean (SD)	Single task Mean (SD)	Dual task Mean (SD)	Single task Mean (SD)	Dual task Mean (SD)	Single task Mean (SD)	Dual task Mean (SD)	Single task Mean (SD)	Dual task Mean (SD)	Single task Mean (SD)	Dual task Mean (SD)
FT (EC)	83.344 (20.293)	84.339 (21.426)	86.677 (22.188)	87.768 (19.137)	88.012 (17.810)	84.830 (17.229)	85.243 (18.411)	81.344 (24.872)	78.101 (24.389)	81.092 (22.518)	83.964 (16.848)	82.836 (22.419)
TR (EC)	83.344 (20.293)	83.821 (19.316)	86.677 (22.188)	84.473 (22.704)	88.012 (17.810)	85.330 (16.876)	85.243 (18.411)	81.506 (22.043)	78.101 (24.389)	80.475 (21.104)	83.964 (16.848)	80.588 (22.819)
TL (EC)	83.344 (20.293)	82.935 (19.814)	86.677 (22.188)	84.261 (20.565)	88.012 (17.810)	82.425 (20.344)	85.243 (18.411)	85.718 (19.569)	78.101 (24.389)	84.317 (16.589)	83.964 (16.848)	81.612 (20.141)
SL-R EC)	83.344 (20.293)	78.693 (24.728)	86.677 (22.188)	83.486 (25.580)	88.012 (17.810)	80.702 (21.287)	85.243 (18.411)	80.602 (23.927)	78.101 (24.389)	79.684 (21.335)	83.964 (16.848)	78.055 (26.083)
SL-L (EC)	83.344 (20.293)	85.513 (19.572)	86.677 (22.188)	83.115 (18.788)	88.012 (17.810)	81.118 (21.363)	85.243 (18.411)	82.374 (20.466)	78.101 (24.389)	80.179 (19.021)	83.964 (16.848)	85.391 (18.733)

**Table 5:** Statistics-counting backward task

Balance tests	Main and interaction effects						
	Three-way interaction	Two-way interactions			Main effects		
	Exp-cond × time × task	Exp-cond × time	Exp-cond × task	Time × task	Exp-cond	Time	Task
FT (EC)	(*F*(2,118) = 0.36, *p* = 0.699, $\eta_{{\text{p}}}^{2}$ = 0.006	(*F*(2,118) = 2.92, *p* = 0.058, $\eta_{{\text{p}}}^{2}$ = 0.047	(*F*(2,118) = 1.51, *p* = 0.226, $\eta_{{\text{p}}}^{2}$ = 0.025	(*F*(1,59) = 0.40, *p* = 0.527, $\eta_{{\text{p}}}^{2}$ = 0.007	(*F*(1.67,98.37) = 3.04, *p* = 0.062, $\eta_{{\text{p}}}^{2}$ = 0.049	(*F*(1,59) = 1.27, *p* = 0.264, $\eta_{{\text{p}}}^{2}$ = 0.021	(*F*(1,59) = 0.12, *p* = 0.728, $\eta_{{\text{p}}}^{2}$ = 0.002
TR (EC)	(*F*(2,118) = 0.38, *p* = 0.69, $\eta_{{\text{p}}}^{2}$ = 0.006	(*F*(2,118) = 2.09, *p* = 0.13, $\eta_{{\text{p}}}^{2}$ = 0.034	(*F*(2,118) = 0.53, *p* = 0.59, $\eta_{{\text{p}}}^{2}$ = 0.009	(*F*(1,59) = 1.82, *p* = 0.18, $\eta_{{\text{p}}}^{2}$ = 0.030	(*F*(1.76,103.93) = 2.80, *p* = 0.07, $\eta_{{\text{p}}}^{2}$ = 0.045	(*F*(1,59) = 0.15, *p* = 0.69, $\eta_{{\text{p}}}^{2}$ = 0.003	(*F*(1,59) = 1.09, *p* = 0.30, $\eta_{{\text{p}}}^{2}$ = 0.018
TL (EC)	(*F*(2,118) = 2.89, *p* = 0.06, $\eta_{{\text{p}}}^{2}$ = 0.047	(*F*(2,118) = 0.24, *p* = 0.79, $\eta_{{\text{p}}}^{2}$ = 0.004	(*F*(2,118) = 1.08, *p* = 0.34, $\eta_{{\text{p}}}^{2}$ = 0.018	(*F*(1,59) = 0.41, *p* = 0.52, $\eta_{{\text{p}}}^{2}$ = 0.007	(*F*(2,118) = 1.85, *p* = 0.16, $\eta_{{\text{p}}}^{2}$ = 0.030	(*F*(1,59) = 1.29, *p* = 0.26, $\eta_{{\text{p}}}^{2}$ = 0.022	(*F*(1,59) = 0.27, *p* = 0.60, $\eta_{{\text{p}}}^{2}$ = 0.005
SL-R (EC)	(*F*(2,118) = 1.49, *p* = 0.23, $\eta_{{\text{p}}}^{2}$ = 0.025	(*F*(2,118) = 1.55, *p* = 0.22, $\eta_{{\text{p}}}^{2}$ = 0.026	(*F*(2,118) = 0.64, *p* = 0.53, $\eta_{{\text{p}}}^{2}$ = 0.011	(*F*(1,59) = 0.24, *p* = 0.63, $\eta_{{\text{p}}}^{2}$ = 0.004	(*F*(1.71,100.64) = 1.59, *p* = 0.21, $\eta_{{\text{p}}}^{2}$ = 0.026	(*F*(1,59) = 1.36, *p* = 0.25, $\eta_{{\text{p}}}^{2}$ = 0.023	(*F*(1,59) = 7.79, *p* = 007*, $\eta_{{\text{p}}}^{2}$ = 0.117
SL-L (EC)	(*F*(2,118) = 1.79, *p* = 0.17, $\eta_{{\text{p}}}^{2}$ = 0.029	(*F*(2,118) = 2.43, *p* = 0.09, $\eta_{{\text{p}}}^{2}$ = 0.040	(*F*(2,118) = 2.18, *p* = 0.12, $\eta_{{\text{p}}}^{2}$ = 0.036	(*F*(1,59) = 0.08, *p* = 0.78, $\eta_{{\text{p}}}^{2}$ = 0.001	(*F*(2,118) = 1.43, *p* = 0.24, $\eta_{{\text{p}}}^{2}$ = 0.024	(*F*(1,59) = 2.20, *p* = 0.14, $\eta_{{\text{p}}}^{2}$ = 0.036	(*F*(1,59) = 1.39, *p* = 0.24, $\eta_{{\text{p}}}^{2}$ = 0.023

*EC* eyes closed, *FT* standing with feet together, *TR* tandem stance with right foot forward, *TL* tandem stance with left foot forward, *SL-R* single leg stance on the right foot, *SL-L* single leg stance on the left foot

## Discussion

The primary aim of this study was to investigate the effect of mental fatigue on single and dual static balance in healthy young adults. To achieve this, participants performed both low and high demanding Stroop tasks (Stroop task_Underload_-number of trials: 1200; mean duration: 43 ± 2.349 min; Stroop task_Overload_-number of trials: 1200; mean duration: 46.6 ± 2.909 min) which served as the mental fatigue conditions in accordance with the existing theories that posit that prolonged cognitive performance could induce mental fatigue due to task underload or overload (Shigihara et al. 2013; Van Cutsem et al. 2022). The control condition entailed watching an engaging but emotionally neutral documentary for 45 min (approximately the same duration with the mental fatigue tasks) as is commonly done in several previous mental fatigue studies (Nikooharf Salehi et al. 2022; Tassignon et al. 2020; Van Cutsem et al. 2022; Verschueren et al. 2020).

Before the discussion on the effect on balance performance, it is important to discuss the findings relating to the successful induction of mental fatigue in the participants. The performance of both Stroop tasks led to significantly higher feeling of subjective mental fatigue in the participants compared to the control condition. This is in line with the findings of previous mental fatigue studies that showed that prolonged performance of Stroop task causes greater feeling of mental fatigue than watching a documentary or reading a magazine for the same duration (Fletcher and Osler 2021; Nikooharf Salehi et al. 2022; Tassignon et al. 2020; Verschueren et al. 2020). However, unlike these previous studies that used Stroop tasks that are 100% incongruent and which may induce fatigue due to task overload (Shigihara et al. 2013), we included a second Stroop task that is 100% congruent as a separate experimental condition to induce mental fatigue in accordance with the underload theory of mental fatigue (Salihu et al. 2022b; Shigihara et al. 2013). The results from the perceived workload using NASA task load index after performance of these tasks suggest that the low and high demanding Stroop tasks might have induced subjective feeling of mental fatigue due to task underload and overload respectively. Essentially, the participants perceived the performance of the high demanding Stroop task to be significantly more effortful and mentally demanding than the control task indicating that the high mental demand of this task might be responsible for the feeling of mental fatigue (Shigihara et al. 2013). On the other hand, the low demanding Stroop task was not perceived by the participants to be more effortful and mentally demanding than watching the documentary. Rather, it was perceived to be more frustrating than the control task indicating that the negative subjective experience of mental fatigue after performing this task may have arisen from the boring and monotonous nature of the task (Shigihara et al. 2013). A previous study by Shigihara et al. (2013) that subjected participants to 30 min of 0-back or 2-back working memory tasks have reported similar findings which they also interpreted as two different types of mental fatigue. In this study, the 0-back task led to increased subjective mental fatigue and sleepiness while only subjective feeling of mental fatigue increased after the more demanding 2-back task causing the authors to conclude that the 0-back and 2-back tasks led to subjective mental fatigue through boredom or underload and excessive mental effort (overload) respectively (Shigihara et al. 2013). Interestingly, the trajectory of the task’s performance overtime in the study by Shigihara and associates lend further support to this interpretation (Shigihara et al. 2013). The performance on the 2-back task remains unchanged over time as the participants applied sufficient effort toward task performance leading to the feeling of fatigue (Shigihara et al. 2013). In contrast, task performance decreased over time in the easy and more boring 0-back session presumably due to under arousal causing subjective mental fatigue and withdrawal of the supervisory attentional system (Pattyn et al. 2008; Shigihara et al. 2013). In an analogous way, our data on the changes in the performance of the Stroop tasks over time have revealed somewhat similar results. We found a significant increase in the number of missed trials with time during the performance of the low demanding Stroop task. Similarly, although not statistically significant, a closer look at the mean scores of the reaction time and number of errors during this task indicate that they both progressively increase across the blocks (time). Alternatively, the reaction time and error rate during the high demanding Stroop task showed an opposite pattern whereby the number of errors during incongruent trials significantly decreased in blocks two and three compared to block one while the reaction times in the same blocks showed a trend towards significant reduction. Both the conflict cost, and conflict adaptation remained unchanged over time in this task suggesting that the feeling of mental fatigue stemmed from application of excessive effort to maintain sufficient level of performance.

Overall, our results indicate that both Stroop tasks used in our study result in subjective mental fatigue. But there was no evidence of objective mental fatigue (decrease in cognitive performance with time-on-task) in the high demanding Stroop task. This is not divergent from the results of similar previous studies. In a study on the effect of mental fatigue on functional performance tests in healthy young adults, Verschueren et al. (2020) did not find a significant change in performance of the Stroop task (100% incongruent) with time over the 90 min duration of the task. Comparable findings were reported in a similar study that used the same type of Stroop task for the same duration in a healthy young population (Tassignon et al. 2020). In the mental fatigue literature, it is obviously more common to observe decreased in performance with time-on-task or objective mental fatigue when the cognitive task is monotonous and intellectually unchallenging than with cognitively demanding tasks (Langner and Eickhoff 2013; Matthews 2011; Moeller et al. 2012; Shigihara et al. 2013). It was suggested that with highly demanding cognitive tasks, participants may apply adequate effort to engage in the task enabling them to maintain performance (Shigihara et al. 2013). The applied mental effort led to subjective feeling of fatigue, but objective performance decrement may only be observed when the time-on-task proceeds for much longer duration than what is required to observe subjective mental fatigue (Ackerman and Kanfer 2009; Shigihara et al. 2013). Therefore, with longer task duration, the high demanding cognitive task in this study and similar previous studies may produce objective mental fatigue in relation to performance decrement with time-on-task. Alternatively, the age of the participants may also play a role as some previous studies in older adults have revealed decrease in performance with time within 30 min of engaging in cognitively demanding tasks such as incongruent Stroop task and executive attention component of attention network test (Holtzer et al. 2010; Nikooharf Salehi et al. 2022). The effect of learning may also play a role in the pattern of result seen in the more demanding Stroop task (Wiehler et al. 2022). Because we define objective mental fatigue in terms of time-on-task effect, any effect of mental fatigue on performance with time might be counteracted by training (learning) effect (Wiehler et al. 2022). Perhaps, if we had assessed objective mental fatigue using a sequential task effect (comparing the performance on a different cognitive task after the experimental and control conditions), we might have observed objective mental fatigue in all the Stroop tasks (Van der Linden et al. 2003).

While our results have revealed a successful induction of mental fatigue in the participants, there was no evidence that their static balance performance was negatively affected in this state. There was no significant difference in either single or dual-task balance between the experimental and control conditions post-intervention. Similarly, the concurrent task (counting backward task) performance during the dual task balance assessment was also not affected indicating that mental fatigue does not impair the available attentional resources for static balance control in the participants. This is in line with a recent study that found no significant difference in static balance control under both single and dual task conditions in healthy young adults after 25 min of performing Stroop task or leisurely reading a magazine (Fletcher and Osler 2021). However, while this study did not report on the concurrent task performance during the dual-task balance assessment, our study included this outcome to have a complete picture of the effect of mental fatigue on attentional resources during dual task static balance in healthy young adults. In another study, static postural sway increased from pre to post in healthy young adults after both 1.5 h of cognitive task and watching documentary for the same duration (Hachard et al. 2020). This finding was attributed to the negative effect of prolonged sitting on subsequent standing balance, not mental fatigue, since the increase in postural sway was observed after sitting both to watch the documentary or carry out the mental fatigue task (Hachard et al. 2020). Therefore, it is reasonable to suggest that within the context of the task durations used in the current and similar previous studies, the induced mental fatigue does not have negative effects on static balance in healthy young adults under both single and dual task conditions. The attentional requirement of maintaining static balance in healthy young adults is minimal compared to the more dynamic challenging balance tasks (Lajoie et al. 1993; Salihu et al. 2022a; Takakusaki 2017). This may explain why healthy young adults can successfully carry out these balance tasks even under conditions of mental fatigue. Mental fatigue is more likely to affect balance control in healthy young adults when the balance task is dynamic and challenging. Indeed, several previous studies have found negative effects of mental fatigue on measures of challenging dynamic balance in this population (Lew and Qu 2014; Qu et al. 2020; Tassignon et al. 2020; Verschueren et al. 2020). For instance, mental fatigue increases the likelihood of loss of balance and decreases the recovery response to unexpected slips and trips during walking in healthy young adults (Lew and Qu 2014; Qu et al. 2020). Another potential explanation for the lack of effect of mental fatigue on static balance in healthy young adults is the absence of any age-related decline in the sensory system in this population (Salihu et al. 2022a). Unlike older adults who may have to rely on attentional resources to control balance even under simple static conditions due to the age-related decline in peripheral sensitivity in the visual, vestibular, and proprioceptive systems (Boisgontier et al. 2012; Glasser and Campbell 1998; Marsh and Geel 2000), young adults have intact peripheral sensitivity and may thus be able to automatically control static balance tasks. In line with this, mental fatigue was found to significantly affect static balance in older adults in a previous study (Fletcher and Osler 2021).

In summary, the performance of both high and low demanding Stroop tasks led to significant feeling of mental fatigue in healthy young adults. However, this did not compromise the attentional resources for static balance control evidenced by lack of effect on both balance and concurrent task performance during dual tasking.

### Limitations

The current study may be limited in several ways and the findings should therefore be interpreted in the context of these limitations. Firstly, we did not assess participant’s level of motivation at the end of the conditioning period (i.e., Stroop/documentary) to test for task (dis)engagement. This in a way limited our conclusion regarding the observed different types of mental fatigue due to underload/overload and should thus be investigated in future studies to provide further evidence for the difference between them. Regarding the lack of effect of mental fatigue on static balance, individual differences in personality and other traits may affect the participants’ response to mentally fatiguing tasks and the subsequent effect on balance. Thus, an analysis of the influence of these characteristics may reveal a sub-group of participants displaying a negative effect of mental fatigue on static balance, which was not investigated in this study. The gender of the participants may also play a role in how they respond to prolonged performance of cognitive tasks both in terms of drop in cognitive performance and balance control. But we did not look at gender difference in the effect of mental fatigue in our participants which is another limitation. Another potential drawback of this study was the limited task duration (45 min) which may be small both from the perspective of inducing objective performance decrement and the typical hours spent on mental tasks in real world situations such as at places of work (for example air traffic controllers and truck drivers on long haul trips) or school. Therefore, it may be interesting to investigate the effect of mental fatigue induced by longer time-on-task on static balance in healthy young adults in future studies. The remaining limitations are related to our balance outcome assessment. We assessed balance using a mobile phone-based balance assessment tool that measured postural sway. However, this might not be as sensitive as other tools of measuring postural sway such as force platforms which give various measures of sway (e.g., sway velocity, sway amplitude, total sway area et cetera) in different directions (antero-posterior and mediolateral). Therefore, we might have missed subtle changes in sway due to mental fatigue that can only be detected in the different parameters obtained from force platform output. Also, the mobile phone-based balance assessment tool we used allowed each balance trial to be conducted for 10 s only. As longer trials and more repeats have been shown to improve the reliability of static balance assessment, the short duration of the balance trials in this study may affect the consistency of the measurement. Last, we did not measure the other component of postural control, i.e., postural muscle tone which may be affected by mental fatigue independent of changes in balance (equilibrium) control. Therefore, we might have missed a potential effect of mental fatigue on this important component of postural control too.

## Conclusion

Within the limit of the time on cognitive task used to induce mental fatigue in the current study, mental fatigue does not seem to negatively affect static balance control in healthy young population. Therefore, dynamic, and more challenging balance tests may be the outcome of choice when investigating mental fatigue related loss of balance and fall risk in the occupational or sport settings in this population. Also, future studies should always consider the controlled/automatic ratio of the chosen balance task based on the age or other clinical characteristics of their participants when investigating this phenomenon.

## Data Availability

All data generated and analysed in this study is available and can be provided if required.
